# The Effects of Acute Deep Seawater Supplementation on Muscle Function after Triathlon

**DOI:** 10.3390/jcm13082258

**Published:** 2024-04-12

**Authors:** Jerónimo Aragón-Vela, Olivia González-Acevedo, Juan Carlos De la Cruz-Márquez, Francisco Javier Rojas Ruíz, Manuel Martínez Marín, Rafael A. Casuso, Julio Plaza-Diaz, Jesus F. Rodriguez Huertas

**Affiliations:** 1 Department of Health Sciences, Area of Physiology, Campus “Las Lagunillas”, Building B3, University of Jaen, 23071 Jaén, Spain; jeroav@ugr.es; 2 Institute of Nutrition and Food Technology “José Mataix”, Biomedical Research Center, University of Granada, 18016 Granada, Spain; nutriol_oga@hotmail.com (O.G.-A.); jhuertas@ugr.es (J.F.R.H.); 3 Department of Physical Education and Sport, Faculty of Sport Sciences, University of Granada, 18071 Granada, Spain; fjrojas@ugr.es (F.J.R.R.); manumar@ugr.es (M.M.M.); 4 Departamento de Ciencias de la Salud, Universidad Loyola Andalucía, 41704 Sevilla, Spain; rcasuso@uloyola.es; 5 Department of Biochemistry and Molecular Biology II, School of Pharmacy, University of Granada, 18071 Granada, Spain; 6 Instituto de Investigación Biosanitaria IBS.GRANADA, Complejo Hospitalario Universitario de Granada, 18014 Granada, Spain; 7 Children’s Hospital of Eastern Ontario Research Institute, Ottawa, ON K1H 8L1, Canada; 8 Department of Physiology, School of Pharmacy, Campus de Cartuja s/n, University of Granada, 18071 Granada, Spain

**Keywords:** exercise physiology, endurance exercise, sweating, hydration

## Abstract

**(1) Background:** Trainers and athletes have always sought to reduce the failure of muscle function during long endurance events. However, nowadays, it is a topic that is generating much debate in the scientific field. Currently, deep-sea water (DSW) intake seems to be a suitable hydration alternative for this type of endurance event. Therefore, the aim of this study was to determine whether DSW consumption during a triathlon event could preserve muscle function after exercise. **(2) Methods:** Nineteen trained male triathletes (age = 39.0 ± 4.25 years; BMI = 23.67 ± 1.81 kg/m^2^) randomly performed three triathlons, one of them consuming DSW (Totum SPORT 30 AB, Laboratories Quinton International, S.L., Spain), the other consuming isotonic placebo and the last with tap water-hydration. A vertical jump test with countermovement and an isometric muscle strength test were conducted before and after the triathlon test. **(3) Results:** There was a significant difference between treatment × time during the isometric muscle strength test. Based on the Tukey post hoc analysis, the peak net force decreased statistically in the placebo (*p* = 0.045) and control conditions (*p* = 0.026), but not in the experimental condition (*p* = 0.121). In addition, all of the conditions studied obtained similar results in the countermovement vertical jump after exercise. **(4) Conclusions:** As a result, consumption of DSW seems to delay the failure of muscle function specifically in isometric exercises but does not improve performance in sports. Thus, DSW does not alter muscle capacity in a negative way; therefore, its consumption may be recommended.

## 1. Introduction

Water is essential for motor action during physical exercise, since it is involved in nerve transmission, nutrient transport, joint lubrication, and muscle contraction [1]. As a result of muscle contractions requiring the use of stored energy through metabolic processes, the body temperature increases. The sweating process accounts for approximately 80% of heat loss [2]. Exercise-induced sweat production is influenced by several factors, including exercise intensity and duration, environmental conditions, and clothing, and can range from extremely low levels to more than 3 L per hour [3]. It is not uncommon to find individuals with unusually salty sweat (>70 mmol/L for sweat sodium concentration) [4,5], even when performing similar cardiorespiratory exercise [6]. Sweat electrolyte concentrations are also highly variable between individuals (e.g., sodium, chloride and potassium). Physical exercise may result in fluid imbalances that could result in a greater instability of osmolality, thereby impairing muscle function [7].

It is important to note that each sport discipline differs in terms of the type and specificity of efforts, energy pathways, exercise durations, and environmental conditions that are encountered during training or competition [8]. In light of this, discipline-specific nutritional recommendations are necessary [8]. Several studies have been conducted regarding nutritional recommendations for team sports such as football/soccer [9,10] (and the sex-based differences within these disciplines) [11,12] or rugby [13]. Endurance athletes such as triathletes may be most at risk for micronutrient deficiency because of low energy consumption, coupled with longer physical activity duration and increased sweat rate [14]. However, it seems that establishing hydration recommendations, especially in triathlon, has been neglected in this aspect.

According to the International Olympic Committee, when exercise duration is ≥2 h, there is high sweat loss; therefore, rehydration during competition recovery should include replacement of water and salts lost through sweat, with sodium included in fluid replacement [15]. Indeed, sodium is the most important osmotically active cation in the extracellular space, and plays a significant role in water balance [16]. After prolonged physical activity, a decrease in serum sodium concentration of less than 135 mmol/L may occur, resulting in hyponatremia [16]. In addition, dehydration is accelerated by hyperthermia, and can lead to glycolysis malfunction. Therefore, a better understanding of muscle functional capacity requires a better understanding of the relationship between dehydration and hyperthermia during exercise and recovery [17].

As a result of voluntary effort, muscle fatigue occurs when the muscle is unable to maintain the required or expected force-generating capacity [18]. The main function of coaches and high-performance athletes is to maintain proper muscle function as long as possible during and after exercise to avoid injury and maintain a high level of performance. In order to support optimal muscle performance, a variety of strategies have been employed. A systematic review suggested that deep-sea water (DSW) consumption may help improve post-exercise muscle functional recovery in humans [19]. Further, Stasiule et al. reported that ingestion of DSW could accelerate recovery of aerobic capacity and leg muscle power when compared with ingestion of water alone [20]. A study has also shown that desalinated ocean mineral water, taken from 662 m below sea level, can accelerate the recovery of aerobic power and lower-body muscle power after a prolonged bout of dehydrating exercise [21]. However, in these studies, dehydration was induced under controlled conditions (laboratory), a situation that does not represent competition conditions in real life. Therefore, research on the effects of DSW consumption on muscle function under competition conditions has not yet been conducted. In addition, the consumption of DSW is a recent supplementation that is gaining popularity among athletes, and has not yet been sufficiently studied to support the benefits it brings, both in terms of performance and muscle capacity.

In addition, sports disciplines with limited access to liquids should be considered. In triathlon, specifically in the swimming and running stages, fluid intake is restricted due to their dynamic nature [22]. The hydration strategies for triathlon have also been studied in order to improve the performance of athletes, since in this sport, there is evidence of recurrent body mass changes caused by sweat rate, fluid concentration changes, and electrolyte loss during competition [23]. In fact, it is not unreasonable to think that a drink containing minerals such as sodium, magnesium, potassium and calcium would be necessary during endurance events, since these are the minerals that are lost through sweating [24]. It is important to note that sodium and magnesium play an important role in the function of nerves and muscles [24]. Potassium contributes to the proper functioning of the heart, nerves, and muscles [24]. The interaction between myosin and actin proteins is enabled by calcium, a key component of muscle contraction [25]. As a result, an efficient hydration strategy must be developed to avoid a significant negative impact on both aerobic performance [26] and athlete health [27] as a result of progressive dehydration [28]. In light of this, our study hypothesis is that DSW contains a significant amount of minerals, which are essential for physical exercise; therefore, its consumption during sports events may preserve muscle functional capacity after exercise. Consequently, the aim of this study was to determine whether DSW consumption during a triathlon event could maintain proper muscle function after exercise. Additionally, it is intended to determine whether consumption of DSW may adversely affect muscle recovery after exercise.

## 2. Materials and Methods

### 2.1. Subjects and Ethics

In the present experimental crossover, placebo-controlled study with three treatment periods, 19 male federated triathletes from different clubs in Andalusia (Spain) participated. Both experimental and placebo treatment periods were completed by these 19 individuals. In spite of this, only 10 of the 19 participants completed the control treatment period. The participants in this study had completed an average of 11.3 ± 5.77 years of training, with a weekly training session schedule of 4.25 ± 1.37 days/week, with an average of 66.25 ± 19.93 km/bike. The inflammatory response data and exclusion criteria have already been described in Acevedo et al. [22]. The participants signed an informed consent form for participation after receiving detailed information about the study’s objectives and procedure, which complied with the ethical standards of the World Medical Association Declaration of Helsinki (2013). It was approved by the local Ethics Committee of the University of Granada (209/CEIH/2018).

The sample size was calculated using G*Power 3.1. software. To make the determination, we selected the following parameters: a moderate effect size (F = 0.4), a significance level of 0.05, a power level of 0.90, and three conditions. The sample size was determined to be at least 15 athletes. In order to ensure the subjects’ success throughout the entire process, 20% more subjects were recruited. Inclusion criteria for the present study were that participants were male, had a weekly triathlon-focused training program, had an annual competitive program, had completed at least three triathlons in the month prior to the study, gave written informed consent, and did not have any metabolic, cardiac, or infectious diseases. They were also required to have extensive experience in triathlon competition. In addition, all participants were required to complete a questionnaire regarding their medical history, previous training experience, and triathlon experience prior to enrollment in the study.

### 2.2. Experimental Design

The experiment was a cross-over, placebo-controlled study with three treatment periods. According to Acevedo et al. [22], the general characteristics of the experimental design have already been published. In brief, the participants performed three complete triathlon tests using its three modalities. During the test, participants were required to swim 800 m (25 m pool), cycle 90 km (net gain of 1100 m of altitude developed in the city of Granada) and run 10 km (outdoor athletics track). The first ampoule was consumed half an hour before the start of the test. All participants were instructed to consume the second ampoule during the transition between swimming and cycling, the third ampoule approximately halfway through cycling, and the fourth ampoule during the transition between cycling and running. Having returned the empty capsules to the researcher, he was able to verify that the capsules had been consumed. This protocol is intended to ensure that electrolyte intake and absorption are balanced throughout the triathlon.

For the purpose of completing the experimental design, participants visited our facilities four times. After consuming a standardized breakfast at least an hour beforehand, participants rested passively for 10 to 15 min before recording their body fat, lean mass, and residual water values. The order of hydration strategies was randomized. A commercial saline product with buffered electrolyte salts was used in the experimental condition (Totum Sport 30 AB, Laboratories Quinton International, S.L., Alicante, Spain). Each participant received three plastic bags containing four translucent drinkable ampoules. In each 10 mL ampoule, 27.297 mg/L sodium, 0.465 mg/L potassium, 19.5 mg/L magnesium and 1.377 mg/L calcium were supplied to the experimental condition. In accordance with Acevedo et al. [22], the seawater was treated. The placebo condition received the same number of drinkable ampoules with identical appearance, but filled with isotonic placebo (cellulose) and 0.9 g/L sodium chloride, while the control condition consumed water ad libitum. The subjects were advised not to engage in strenuous exercise 72 h prior to each protocol. In summary, the control condition consumed tap water ad libitum, the experimental condition consumed DSW, and the placebo condition consumed isotonic solution (Table 1). The study was conducted at the University of Granada’s Faculty of Sports Science.

### 2.3. Experimental Trial

In order to increase muscle and liver glycogen stores, a strictly controlled diet was used. Participants were instructed to consume at least 8.5 g of carbohydrate per kilogram of body weight and at least 50 mL/kg of fluid 72 h prior to the test [29]. Additionally, we asked them to refrain from consuming any sources of caffeine or alcohol. VO_2_max was determined at the first visit using a graded exercise test to exhaustion on an electronic brake (Lode Excalibur sports ergometer, Groningen, The Netherlands), with inspired and expired oxygen and carbon dioxide fractions measured breath by breath. The graded exercise protocol was initiated at 150 W and increased by 30 W × 2 min^−1^ until voluntary exhaustion or pedaling frequency lower than 75 rpm has been reached [30].

As previously described [3,6] on a force platform (Dinascan/IBV, Biomechanics Institute of Valencia, Spain at 500 Hz), del Coso et al. used the CVJ to determine maximum height, peak strength, peak power, and body mass [6]. Prior to the jump test, participants performed 10 min of running, dynamic leg exercises, and jumping drills, and they were instructed to bend their knees and jump as high as possible, keeping their hands on their hips and landing on both feet [6]. A force platform and a barbell loaded supra-maximally (170 kg) were used to measure IMS. During this measurement, participants were trained in advance to stand with the barbell at the height of the anterior tibia spine, bending their knees about 135° and extending their upper limbs fully extended [3]. The participants were instructed to pull maximally for 3 s using their whole body (mainly their lower and upper limbs). Throughout the test, verbal motivation was provided to maintain this position. During the performance, an adjustable lumbar-posterior protector was used to provide support and protection. Subsequently, the triathlon test was conducted. Prior to the triathlon test, participants drank 500 mL of tap water. Participants were allowed to consume water and food at their discretion during the test, repeating the same pattern each time. As for the environmental conditions, a weather station located in the departure laboratory and validated by the Observatory of Meteorology and Astronomy (Indoor BTH Weather Station Autonautic 72 mm, Autonautic Instrumental S.L., El Masnou, Barcelona, Spain) of the University of Granada was used. As for the pool temperature, it was 25 degrees in all three sporting events.

Finally, as this compound is relatively new and it is not known whether it can cause any adverse effects, participants were instructed to report any incidents that occurred during ingestion of the vials and any side effects related to their consumption. In addition, a 0–10 rating of the perceived exertion scale (RPE) was recorded immediately after the cessation of each stage. Once the triathlon is completely finished and after having taken a 5 min rest, individuals performed the CVJ and IMS tests again, as described by del Coso et al. [3,6]. The participants were then asked to complete a detailed questionnaire regarding their fluid intake for statistical analysis. On the third visit, which took place within a period of 7–14 days, the same triathlon test was performed, but with a different hydration strategy. Finally, the control condition conducted a third trial in which only tap water was consumed ad libitum.

### 2.4. Anthropometric and Strength Variables

According to Acevedo et al. [22], the system of anthropometric measurements and the maximum oxygen consumption were conducted. In running clothes, participants were weighed with a segmental bioelectrical impedance analyzer (InBody 720) (Biospace Inc., Seoul, Republic of Korea) fifteen minutes prior to the triathlon test and one minute after the end of the running test, in order to establish the general anthropometric data. Pre- and post-body mass measurements were used to calculate body mass loss (pre-body mass − post-body mass/pre-exercise body mass) × 100 [31]. Participants in all three triathlon events were weighed in the same clothes and without shoes. A Quattro Jump force platform (Kistler, Winterthur, Switzerland) was used for the strength variables (CVJ and isometric strength). A lumbar-posterior protector (Freetoo, Guangdong, China), a 20 kg barbell, and six discs of 25 kg (Proiron, Shanxi Regent Works Inc., Taiyuan, China) were also used for the isometric evaluation. Additionally, the data extracted from the force platform was calculated using Microsoft Office 2010 (Excel).

### 2.5. Statistical Analysis

In this study, the data are expressed as mean ± standard error of the mean. An assessment of the normality of the distribution was made using the Shapiro–Wilk test. For the purpose of analyzing homogeneity of variance, the Levene test was used. In order to examine the effects of time, treatment, and the interaction between them (time × treatment), we employed a linear mixed model to analyze the differences effects of variables in control, placebo, and experimental conditions, as well as the differences between pre-exercise and post-exercise differences between the conditions. As a result of using the lme4 package [32] in the R program, the linear mixed model was conducted. Since it employs modern, efficient linear algebra methods, such as those implemented in the Eigen package, as well as reference classes to prevent undue copying of large objects, the lme4 package is likely to be faster and more memory-efficient than other programs. Additionally, the software is capable of constructing generalized linear mixed models using the glmer function, so as to maximize the amount of information available when loss patients are included in some of the analyzed conditions [32]. Our post hoc analysis was performed using R program version 3.6 and the emmeans function using the Tukey method. The level of statistical significance was set at *p* < 0.05. The statistical analysis was conducted using SPSS/PC version 22 (SPSS Inc., Chicago, IL, USA), and R program v. 3.6.

## 3. Results

### Participants’ Anthropometric and Functional Characteristics

As shown in Table 2, participants’ ages, anthropometric characteristics, and peak oxygen consumption are provided. There were no differences in baseline characteristics between the conditions compared. In addition, Table 3 provides information regarding the environmental conditions, changes in body weight, perception of effort, and performance of each condition at different stages of the test. Comparison of the times of each transmission between experimental, control, and placebo conditions revealed no significant differences in performance. There were no significant differences in weight before and after the triathlon. Based on the values of perceived exertion, there were no significant differences between the different transition stages. Observing the temperature data, it is evident that the three triathlon events began with similar temperatures on all three occasions and ended with similar temperatures.

In Table 4, the results of the CVJ test and the IMS test are presented. According to the linear mixed model of the CVJ test, take-off vertical velocity and peak relative power differed depending on the time period and the conditions. Peak relative power and peak net force were affected by treatment. The interaction between treatment × time, however, did not show any statistical changes. On the basis of the IMS test, the linear mixed model showed significant differences between treatments for peak relative power and peak net force. On the basis of the IMS test, we found an interaction between treatment and time in peak net force, and the Tukey post hoc analysis showed that peak net force decreased statistically in the placebo and control conditions as compared to the experimental condition.

## 4. Discussion

The aim of this study was to determine whether DSW consumption during a triathlon event could maintain proper muscle function after exercise. The main outcomes of this were that DSW consumption dampens post-exercise loss muscle function specifically in isometric exercises. Indeed, the ISM test showed that the maximal net force decreased statistically in the placebo and control conditions, while in the experimental condition, no such differences were shown. However, it does not improve the CVJ test and performance.

The results obtained with the CVJ test showed that the values of the peak net force values appear to be lower at baseline than at post, which is quite remarkable, since the post values were obtained after an endurance event lasting more than two hours; however, no significant interaction between treatment and time was observed. Different authors argue that, in order to prevent these occurrences with peak net force, it is necessary to warm up more efficiently in order to reach the maximum level of jump [33]. With regard to the results obtained in the ISM test, the linear mixed model, and the interaction treatment x time, and after a post hoc analysis, the placebo and control condition had a statistically significant reduction in peak force, which means that the placebo and control condition could not maintain proper muscle function after exercise, showing values that were significantly lower than pre-exercise values. This may be due to the fact that sodium chloride consumption in isolation has no effect on muscle recovery, given that the placebo condition has similar behavior to the control condition. Indeed, an exclusive consumption of water is not enough to maintain optimal muscle performance during long-duration sport events [34]. However, the experimental condition showed no significant differences with respect to the pre-exercise values. This lack of significance indicates that the experimental condition achieved the same isometric strength values both at the beginning and at the end of the triathlon test. Therefore, DSW consumption could have maintained a muscle capacity comparable to the beginning of the triathlon test, while placebo and control conditions did not.

This discrepancy between CVJ and ISM results may be explained by the fact that jumping exercises rely heavily on reflex stretching, whereas isometric exercises rely heavily on neural activation [35,36]. DSW consists primarily of sodium, potassium, calcium, and magnesium, whose main functions are to improve muscle contraction and nerve impulse transmission [19,24,37]. The fact that most of the minerals in DSW are associated with improving neuromuscular communication and contraction explains why DSW has performed better than placebo and control in the ISM test. However, in the CVJ test, given that DSW supplementation was not involved in the improvement of the stretch reflex, it is understandable that no improvement was obtained compared to the placebo and control conditions. As a result, DSW consumption appears to positively affect muscle function, because it is capable of achieving the same post-exercise isometric strength values as the placebo and control group. Furthermore, it has been established that DSW consumption does not adversely affect the muscular performance of endurance athletes.

During exercise, hyperthermia and dehydration may both contribute to increased glycogenolysis and decreased glycogen resynthesis [38]. Although core and muscle hyperthermia are believed to contribute to glycogen impairments following high-intensity or prolonged exercise (internal hyperthermia) or exercising in hot environments (external hyperthermia), it is closely related to dehydration [17]. To optimize long-term performance, it is essential to maintain an appropriate body temperature and to consume adequate fluids during and after exercise. In light of the obvious practical applications, further studies are needed to understand the effects of hyperthermia and dehydration on glycogen metabolism.

Even though the participants lost more than 2% of their weight due to dehydration, they were able to maintain optimal performance, which enabled them to complete the three tests in similar time and RPE values. According to Casa et al. [39], dehydration values of 3% to 4% may adversely affect jumping and strength performance, which would be in agreement with our results, as it is the placebo and control conditions that show significant differences in the isometric test, indicating that the initial values are different from those reached in the posttest. However, the experimental condition, despite having a higher degree of dehydration than the placebo and control conditions, did not affect their functional capacity in the isometric exercises, possibly due to the consumption of deep seawater. In endurance events, recommending that athletes’ fluid intake should be based on their thirst almost entirely prevented the development of hyponatremia, without inducing clinically significant hypernatremia or negatively affecting race completion time [40].

In various studies [1,20,21,41,42], DSW consumption has been shown to improve performance and reduce muscle fatigue. However, these data partially contradict the results obtained, as it appears to increase post-exercise muscle performance over time, but there is no improvement in sports performance. This is possibly due to the fact that previous studies were conducted in a controlled climate in which dehydration was induced. Indeed, none of the studies indicated that food was consumed, so participants consumed only beverages provided by the researchers. The results of this study may better demonstrate the effects of beverages, but they do not reflect the reality of endurance athletes, who often supplement with food and liquids during competition, as was done in this study. Indeed, if one looks at the results obtained in the study by Olivia-Acevedo et al., where the nature of the competition itself was taken into consideration, with food and fluid intake ad libitum, the consumption of DSW did not improve sports performance [22]. However, it should be noted that the consumption of DSW stimulates myokine production, which may contribute to delaying the failure of muscle function after exercise, but not to improved performance [22].

It should be noted that the multiple ingredients in the saline drink studied, as well as in several dietary supplements on the market, make it difficult to determine the effects and safety of these products. According to Casazza et al. [43], it is essential to obtain information regarding all the ingredients of the product prior to its use by athletes. Many products that claim to be beneficial for performance contain proprietary blends of ingredients without information on the amount of each ingredient.

### Limitations and Strength

There are a number of limitations to this study: (i) as our objectives were more concerned with the hydration capacity of DSW consumption than with the triathlon modality itself, the triathlon modality used is not within the official modalities imposed by the Spanish Triathlon Federation; and (ii) the control condition did not reach the same number of subjects as the experimental condition and placebo, which could affect the validity and reliability of the data obtained. In order to minimize the problems with compliance of the participants in the control condition, a linear mixed model was used. In a linear mixed model, information is maximized differently than in a conventional analysis of variance test. It is used specifically in statistical cases involving loss of participants.

Nevertheless, the major strength of this study is that it opens up a new area of research, in which DSW consumption could be used as a sports supplement without causing negative effects on muscle recovery. This study also represents a real-life scenario for a triathlon event, in which participants may consume food and beverages ad libitum and be exposed to a variety of weather conditions, including wind, temperature, and humidity. Future studies will certainly be of interest to extend this knowledge to other, more severe physiological conditions, such as hypoxic environments, as well as to determine whether deep water consumption alleviates the mitochondrial alterations caused by moderate hypoxia at the muscular level.

In terms of practical application, we confirm the benefit of DSW consumption in delaying muscle function failure. Therefore, its consumption may be ideal in those training sessions where the volume and load are demanding. In this way, the triathlete could maintain a high level of performance over time and, in turn, could prevent the probability of suffering some kind of overload that could lead to some kind of injury. In addition, an improvement in muscle function might help the triathlete to face the next training session in a better condition, especially in master triathletes, who do not have the same muscle recovery as younger triathletes.

## 5. Conclusions

The consumption of DSW seems to delay the failure of muscle function specifically in isometric exercises; however, it does not improve the performance in triathlons. In addition, DSW consumption does not generate problems that interfere negatively with muscle fatigue, so its consumption can be recommended. The present study may serve as evidence that field tests often do not coincide with laboratory tests, and provides relevant information for athletes, coaches and nutritionists to know the implications of the use of commercially available saline beverages during endurance competitions.

## Figures and Tables

**Table 1:** Beverage composition.

Experimental	Placebo	Control
27.297 mg/L sodium	0.9 g/L sodium chloride	Tap water
0.465 mg/L potassium		
19.5 mg/L magnesium		
1.377 mg/L calcium		

**Table 2:** Characteristics of participants.

Variables	Control (*n* = 10)	Experimental (*n* = 19)	Placebo (*n* = 19)	*p*-Value
Age (years)	40.7 ± 5.89	39.15 ± 5.28	39.15 ± 5.28	n.s.
Body height (cm)	176.6 ± 8.04	176.89 ± 6.08	176.89 ± 6.08	n.s.
Body mass (Kg)	71.92 ± 13.32	75.2 ± 11.03	75.72 ± 10.95	n.s.
Total Body Water (Kg)	46.54 ± 6.58	46.2 ± 4.84	47.4 ± 5.32	n.s.
Protein (kg)	12.72 ± 1.84	12.42 ± 1.31	12.98 ± 1.39	n.s.
Body Fat Mass (kg)	7.2 ± 3.92	8.74 ± 3.86	8.22 ± 4.07	n.s.
Body Mass Index (kg/m^2^)	22.58 ± 2.51	23.27 ± 2.27	23.49 ± 1.81	n.s.
VO_2_max (mL/kg/min)	45.31 ± 9.52	41.5 ± 11.32	41.5 ± 11.32	n.s.

Results are expressed as mean ± standard deviation; n.s., not significant.

**Table 3:** Time, body mass, rate of perceived exertion, and temperature of the test.

	Control Condition	Placebo Condition	Experimental Condition	*p*-Value
Swimming time (Min)	14.69 ± 1.41	14.76 ± 1.39	14.67 ± 1.2	n.s.
Cycling time (Min)	157.2 ± 8.02	167.21 ± 12.04	166.68 ± 11.79	n.s.
Running time (Min)	48.61 ± 6.46	51.58 ± 8.69	52.23 ± 7.2	n.s.
Complete test time (Min)	220.2 ± 14.39	233.56 ± 19.65	233.59 ± 18.09	n.s.
Pre-Body Mass (kg)	73.08 ± 12.3	74.47 ± 12	75.77 ± 11.81	n.s.
Post-Body Mass (kg)	70.48 ± 11.64	71.57 ± 11.62	72.59 ± 11.56	n.s.
Weight loss percentage	−3.55 ± 1.51	−3.89 ± 2.17	−4.19 ± 2.7	n.s
Swimming RPE	7.94 ± 0.93	7.03 ± 0.65	6.72 ± 0.65	n.s.
Cycling RPE	8.4 ± 0.54	7.78 ± 0.41	8.02 ± 1.26	n.s.
Running RPE	8.87 ± 0.54	9.01 ± 0.8	9.13 ± 0.85	n.s.
Initial temperature (°C)	16.3 ± 1.16	15.21 ± 7.27	16.21 ± 6.63	n.s.
Final temperature (°C)	28.7 ± 1.88	27.05 ± 8.07	29.49 ± 7.37	n.s.

Results are expressed as mean ± standard deviation. RPE, rate of perceived exertion; n.s., not significant.

**Table 4:** Testing the strength and power of muscles by countermovement vertical jump and isometric muscles.

**Countermovement Vertical Jump Test**	**Control Condition**		**Placebo Condition**		**Experimental Condition**		***p*-Value**		
	**Pre**	**Post**	**Pre**	**Post**	**Pre**	**Post**	**Time (T)**	**Treatment (t)**	**T × t**
Flight Time (s)	0.48 ± 0.07	0.51 ± 0.04	0.50 ± 0.1	0.49 ± 0.06	0.48 ± 0.09	0.48 ± 0.06	0.619	0.925	0.887
Jump height (m)	0.30 ± 0.07	0.32 ± 0.06	0.33 ± 0.1	0.31 ± 0.06	0.30 ± 0.09	0.30 ± 0.06	0.349	0.996	0.982
Take-off vertical velocity (m/s)	2.1 ± 0.4	2.4 ± 0.4	2.2 ± 0.3	2.4 ± 0.5	2.7 ± 1.2	2.5 ± 0.8	0.024	0.736	0.905
Peak relative power (PP) (W/Kg)	21.0 ± 5.9	27.4 ± 3.9	22.0 ± 5.0	27.8 ± 6.8	27.6 ± 9.7	29.1 ± 8.7	0.019	0.027	0.705
Peak net force (N)	881.0 ± 261.5	1026.2 ± 276.8	1009.2 ± 312.4	1106.9 ± 276.6	962.8 ± 253.2	1099.5 ± 233.6	0.277	0.012	0.467
**Isometric Muscle Strength Test**	**Control Condition**		**Placebo Condition**		**Experimental Condition**		***p*-Value**		
	**Pre**	**Post**	**Pre**	**Post**	**Pre**	**Post**	**Time (T)**	**Treatment (t)**	**T × t**
Peak relative power (PP) (W/Kg)	72.4 ± 44.2	47.5 ± 34.3	71.0 ± 103.0	64.2 ± 87.3	80.9 ± 97.9	51.8 ± 48.7	0.402	0.003	0.901
Peak net force (N)	764.2 ± 271.6	616.8 ± 280.3 ^a^	742.8 ± 195.3	604.3 ± 131.6 ^a^	734.7 ± 253.2	675.0 ± 164.2 ^b^	0.093	0.043	0.046

Results are expressed as mean ± standard deviation. *p*-values were determined for time and treatment × time, and different letters mean significant differences (*p* < 0.05) and were calculated with Tukey post hoc multiple comparisons for observed means.

## Data Availability

All data needed to evaluate the conclusions in the paper are present in the paper.

## References

[1] Harris P.R., Keen D.A., Constantopoulos E., Weninger S.N., Hines E., Koppinger M.P., Khalpey Z.I., Konhilas J.P. (2019). Fluid type influences acute hydration and muscle performance recovery in human subjects. J. Int. Soc. Sports Nutr..

[2] Nuccio R.P., Barnes K.A., Carter J.M., Baker L.B. (2017). Fluid Balance in Team Sport Athletes and the Effect of Hypohydration on Cognitive, Technical, and Physical Performance. Sports Med..

[3] Del Coso J., Gonzalez-Millan C., Salinero J.J., Abian-Vicen J., Soriano L., Garde S., Perez-Gonzalez B. (2012). Muscle damage and its relationship with muscle fatigue during a half-iron triathlon. PLoS ONE.

[4] Brown M.B., Haack K.K., Pollack B.P., Millard-Stafford M., McCarty N.A. (2011). Low abundance of sweat duct Cl-channel CFTR in both healthy and cystic fibrosis athletes with exceptionally salty sweat during exercise. Am. J. Physiol. Regul. Integr. Comp. Physiol..

[5] Del Coso J., Gonzalez-Millan C., Salinero J.J., Abian-Vicen J., Areces F., Lledo M., Lara B., Gallo-Salazar C., Ruiz-Vicente D. (2016). Effects of oral salt supplementation on physical performance during a half-ironman: A randomized controlled trial. Scand. J. Med. Sci. Sports.

[6] Del Coso J., Gonzalez C., Abian-Vicen J., Salinero Martin J.J., Soriano L., Areces F., Ruiz D., Gallo C., Lara B., Calleja-Gonzalez J. (2014). Relationship between physiological parameters and performance during a half-ironman triathlon in the heat. J. Sports Sci..

[7] Edwards A.M., Noakes T.D. (2009). Dehydration: Cause of fatigue or sign of pacing in elite soccer?. Sports Med..

[8] Nowaczyk P.M., Adamczewski J., Durkalec-Michalski K. (2023). Practical Application and Methodological Considerations on the Basics of Sports Nutrition in Basketball: A Comprehensive Systematic Review of Observational and Interventional Studies. Nutrients.

[9] Collins J., Maughan R.J., Gleeson M., Bilsborough J., Jeukendrup A., Morton J.P., Phillips S., Armstrong L., Burke L.M., Close G.L. (2021). UEFA expert group statement on nutrition in elite football. Current evidence to inform practical recommendations and guide future research. Br. J. Sports Med..

[10] Oliveira C.C., Ferreira D., Caetano C., Granja D., Pinto R., Mendes B., Sousa M. (2017). Nutrition and supplementation in soccer. Sports.

[11] Danielik K., Książek A., Zagrodna A., Słowińska-Lisowska M. (2022). How Do Male Football Players Meet Dietary Recommendations? A Systematic Literature Review. Int. J. Environ. Res. Public Health.

[12] Dobrowolski H., Karczemna A., Włodarek D. (2020). Nutrition for female soccer players—Recommendations. Medicina.

[13] Carruthers J. (2016). Nutritional Practices, Interventions and recommendations for junior rugby league players. Sports Nutr. Ther..

[14] Logue D., Madigan S.M., Delahunt E., Heinen M., Mc Donnell S.J., Corish C.A. (2018). Low Energy Availability in Athletes: A Review of Prevalence, Dietary Patterns, Physiological Health, and Sports Performance. Sports Med..

[15] (2011). IOC consensus statement on sports nutrition 2010. J. Sports Sci..

[16] Arnaoutis G., Anastasiou C.A., Suh H., Maraki M., Tsekouras Y., Dimitroulis E., Echegaray M., Papamichalopoulou D., Methenitis S., Sidossis L.S. (2020). Exercise-Associated Hyponatremia during the Olympus Marathon Ultra-Endurance Trail Run. Nutrients.

[17] Lopez-Torres O., Rodriguez-Longobardo C., Escribano-Tabernero R., Fernandez-Elias V.E. (2023). Hydration, Hyperthermia, Glycogen, and Recovery: Crucial Factors in Exercise Performance-A Systematic Review and Meta-Analysis. Nutrients.

[18] Kataoka R., Vasenina E., Hammert W.B., Ibrahim A.H., Dankel S.J., Buckner S.L. (2022). Is there Evidence for the Suggestion that Fatigue Accumulates Following Resistance Exercise?. Sports Med..

[19] Aragon-Vela J., Gonzalez-Acevedo O., Plaza-Diaz J., Casuso R.A., Huertas J.R. (2022). Physiological Benefits and Performance of Sea Water Ingestion for Athletes in Endurance Events: A Systematic Review. Nutrients.

[20] Stasiule L., Capkauskiene S., Vizbaraite D., Stasiulis A. (2014). Deep mineral water accelerates recovery after dehydrating aerobic exercise: A randomized, double-blind, placebo-controlled crossover study. J. Int. Soc. Sports Nutr..

[21] Hou C.W., Tsai Y.S., Jean W.H., Chen C.Y., Ivy J.L., Huang C.Y., Kuo C.H. (2013). Deep ocean mineral water accelerates recovery from physical fatigue. J. Int. Soc. Sports Nutr..

[22] Acevedo O.G., Aragon-Vela J., De la Cruz Marquez J.C., Marin M.M., Casuso R.A., Huertas J.R. (2022). Seawater Hydration Modulates IL-6 and Apelin Production during Triathlon Events: A Crossover Randomized Study. Int. J. Environ. Res. Public Health.

[23] Pahnke M.D., Trinity J.D., Zachwieja J.J., Stofan J.R., Hiller W.D., Coyle E.F. (2010). Serum sodium concentration changes are related to fluid balance and sweat sodium loss. Med. Sci. Sports Exerc..

[24] American College of Sports M., Sawka M.N., Burke L.M., Eichner E.R., Maughan R.J., Montain S.J., Stachenfeld N.S. (2007). American College of Sports Medicine position stand. Exercise and fluid replacement. Med. Sci. Sports Exerc..

[25] Rodrigues P., Trajano G.S., Stewart I.B., Minett G.M. (2022). Potential role of passively increased muscle temperature on contractile function. Eur. J. Appl. Physiol..

[26] Sawka M.N., Noakes T.D. (2007). Does dehydration impair exercise performance?. Med. Sci. Sports Exerc..

[27] Goulet E.D.B., Hoffman M.D. (2019). Impact of Ad Libitum Versus Programmed Drinking on Endurance Performance: A Systematic Review with Meta-Analysis. Sports Med..

[28] Gonzalez-Alonso J., Mora-Rodriguez R., Below P.R., Coyle E.F. (1997). Dehydration markedly impairs cardiovascular function in hyperthermic endurance athletes during exercise. J. Appl. Physiol..

[29] Rowlands D.S., Houltham S.D. (2017). Multiple-Transportable Carbohydrate Effect on Long-Distance Triathlon Performance. Med. Sci. Sports Exerc..

[30] Bejder J., Andersen A.B., Buchardt R., Larsson T.H., Olsen N.V., Nordsborg N.B. (2017). Endurance, aerobic high-intensity, and repeated sprint cycling performance is unaffected by normobaric “Live High-Train Low”: A double-blind placebo-controlled cross-over study. Eur. J. Appl. Physiol..

[31] Deshayes T.A., Jeker D., Goulet E.D.B. (2020). Impact of Pre-exercise Hypohydration on Aerobic Exercise Performance, Peak Oxygen Consumption and Oxygen Consumption at Lactate Threshold: A Systematic Review with Meta-analysis. Sports Med..

[32] Wang T., Graves B., Rosseel Y., Merkle E.C. (2022). Computation and application of generalized linear mixed model derivatives using lme4. Psychometrika.

[33] Boullosa D., Del Rosso S., Behm D.G., Foster C. (2018). Post-activation potentiation (PAP) in endurance sports: A review. Eur. J. Sport Sci..

[34] Williamson E. (2016). Nutritional implications for ultra-endurance walking and running events. Extrem. Physiol. Med..

[35] Desbrosses K., Babault N., Scaglioni G., Meyer J.P., Pousson M. (2006). Neural activation after maximal isometric contractions at different muscle lengths. Med. Sci. Sports Exerc..

[36] Van Hooren B., Zolotarjova J. (2017). The Difference between Countermovement and Squat Jump Performances: A Review of Underlying Mechanisms with Practical Applications. J. Strength Cond. Res..

[37] Jeukendrup A., Gleeson M. (2019). Sport Nutrition.

[38] Logan-Sprenger H.M., Heigenhauser G.J., Jones G.L., Spriet L.L. (2013). Increase in skeletal-muscle glycogenolysis and perceived exertion with progressive dehydration during cycling in hydrated men. Int. J. Sport Nutr. Exerc. Metab..

[39] Casa D.J., Cheuvront S.N., Galloway S.D., Shirreffs S.M. (2019). Fluid Needs for Training, Competition, and Recovery in Track-and-Field Athletes. Int. J. Sport Nutr. Exerc. Metab..

[40] Lopez de Lara D., Ruiz-Sanchez J.G., Cuesta M., Seara G., Calle-Pascual A.L., Rubio Herrera M.A., Runkle I., Verbalis J.G. (2021). Exercise-Induced Hyponatremia: An Assessment of the International Hydration Recommendations Followed During the Gran Trail De Penalara and Vitoria-Gasteiz Ironman Competitions. Front. Nutr..

[41] Keen D.A., Constantopoulos E., Konhilas J.P. (2016). The impact of post-exercise hydration with deep-ocean mineral water on rehydration and exercise performance. J. Int. Soc. Sports Nutr..

[42] Perez-Turpin J.A., Trottini M., Chinchilla-Mira J.J., Cyganik W. (2017). Effects of seawater ingestion on lactate response to exercise in runners. Biol. Sport.

[43] Casazza G.A., Tovar A.P., Richardson C.E., Cortez A.N., Davis B.A. (2018). Energy Availability, Macronutrient Intake, and Nutritional Supplementation for Improving Exercise Performance in Endurance Athletes. Curr. Sports Med. Rep..

